# Spatial rank-based multifactor dimensionality reduction to detect gene–gene interactions for multivariate phenotypes

**DOI:** 10.1186/s12859-021-04395-y

**Published:** 2021-10-04

**Authors:** Mira Park, Hoe-Bin Jeong, Jong-Hyun Lee, Taesung Park

**Affiliations:** 1 Department of Preventive Medicine, Eulji University, Daejeon, 34824 Republic of Korea; 2 Department of Statistics, Korea University, Seoul, 02841 Republic of Korea; 3 Department of Statistics, Seoul National University, Seoul, 08826 Republic of Korea

**Keywords:** Fuzzy clustering, Gene–gene interaction, Multifactor dimensionality reduction, Spatial rank statistic

## Abstract

**Background:**

Identifying interaction effects between genes is one of the main tasks of genome-wide association studies aiming to shed light on the biological mechanisms underlying complex diseases. Multifactor dimensionality reduction (MDR) is a popular approach for detecting gene–gene interactions that has been extended in various forms to handle binary and continuous phenotypes. However, only few multivariate MDR methods are available for multiple related phenotypes. Current approaches use Hotelling’s T^2^ statistic to evaluate interaction models, but it is well known that Hotelling’s T^2^ statistic is highly sensitive to heavily skewed distributions and outliers.

**Results:**

We propose a robust approach based on nonparametric statistics such as spatial signs and ranks. The new multivariate rank-based MDR (MR-MDR) is mainly suitable for analyzing multiple continuous phenotypes and is less sensitive to skewed distributions and outliers. MR-MDR utilizes fuzzy k-means clustering and classifies multi-locus genotypes into two groups. Then, MR-MDR calculates a spatial rank-sum statistic as an evaluation measure and selects the best interaction model with the largest statistic. Our novel idea lies in adopting nonparametric statistics as an evaluation measure for robust inference. We adopt tenfold cross-validation to avoid overfitting. Intensive simulation studies were conducted to compare the performance of MR-MDR with current methods. Application of MR-MDR to a real dataset from a Korean genome-wide association study demonstrated that it successfully identified genetic interactions associated with four phenotypes related to kidney function. The R code for conducting MR-MDR is available at https://github.com/statpark/MR-MDR.

**Conclusions:**

Intensive simulation studies comparing MR-MDR with several current methods showed that the performance of MR-MDR was outstanding for skewed distributions. Additionally, for symmetric distributions, MR-MDR showed comparable power. Therefore, we conclude that MR-MDR is a useful multivariate non-parametric approach that can be used regardless of the phenotype distribution, the correlations between phenotypes, and sample size.

## Background

Many attempts have been made to identify susceptible genes that influence the risk of complex diseases such as autism, hypertension, and diabetes [1–3]. Analyzing a single locus is not enough to understand the pathophysiology of complex diseases and results in the so-called missing heritability problem. To overcome this problem, several studies have sought to identify gene–gene interactions (GGIs) or gene-environmental interactions [4–6].

As a non-parametric model-free approach, multifactor dimensionality reduction (MDR) has been widely applied for detecting GGIs [5]. For binary phenotypes, such as those analyzed in case–control studies, MDR divides high-dimensional genotype combinations into a one-dimensional variable with two groups (high-risk and low-risk), according to whether the ratio of cases to controls exceeds a threshold. Then it finds the interaction model that best predicts the disease status. Balanced accuracy can be used for an evaluation measure [7]. To prevent overfitting, k-fold cross-validation (CV) can be applied. Cross-validation consistency (CVC), or the number of times each single-nucleotide polymorphism (SNP) combination is chosen as best, is obtained during the k-fold CV process. The SNP combination with the highest CVC is defined as the final best interaction model [8]. MDR has several advantages: i) the dimensions of the data are effectively reduced, ii) no specific genetic model is assumed, and iii) high-order interactions can be identified, even if there are no significant main effects [9, 10].

Since its introduction, many studies have been conducted to broaden the scope of application of MDR. According to Gola et al. [4], about 800 MDR-related studies were found as of February 2014 when searching PubMed and Google Scholar. For discrete traits, log-linear models MDR and robust MDR have been proposed to handle data with empty or sparse cells [11, 12]. Odds ratio MDR was proposed, replacing the naïve classifier with the odds ratio [9] and optimal MDR replaced the fixed threshold with a data-driven threshold using an ordered combinatorial partitioning algorithm [13]. As a method of dealing with continuous traits, generalized MDR (GMDR) was proposed. GMDR can handle both dichotomous and continuous phenotypes and can adjust for covariates [14]. Quantitative MDR (QMDR) for continuous traits uses the sample mean of each genotype combination as a classifier, reducing the computing time with comparable performance [15]. Recently, cluster-based MDR (CL-MDR) has been proposed as a method that is less sensitive to outliers and distributional assumptions [16]. For survival time with censored data, Surv-MDR was proposed, which uses the log-rank test statistic to classify the cells of a multifactor combination [17]. Cox-MDR and accelerated failure time MDR are extended versions of GMDR for the survival phenotype based on Cox regression and the accelerated failure time model, respectively [18, 19]. Recently, Kaplan–Meier MDR was also developed, which uses the median Kaplan–Meier survival time as a classifier [20].

As described above, many studies have been conducted to identify genetic interactions associated with single phenotype, but only a few studies have been done on methodologies to treat multiple phenotypes. For complex diseases, several correlated traits are often measured together. For example, weight, the waist-hip ratio, and the body mass index (BMI) can be jointly measured as obesity-related traits. The power to detect associations between genes and these traits is expected to increase if the multivariate approach is adopted [21]. Therefore, more research on multivariate methods detecting GGIs is needed. Recently, multivariate generalized MDR (GEE-GMDR) extended GMDR to the multivariate case by constructing generalized estimation equation models [22]. GEE-GMDR provides stable results, but it does not always show higher power than GMDR [23]. Multivariate QMDR (multi-QMDR) extended QMDR using principal component analysis scores and Hotelling’s T^2^ statistic as a classifier and an evaluation measure instead [23]. Multi-QMDR gives a high CVC and stable results, but it is prone to outliers or influencing points. More recently, multivariate cluster-based MDR (multi-CMDR) has been proposed [24]. Multi-CMDR applies fuzzy k-means clustering to discriminate between high- and low-risk groups and uses Hotelling’s T^2^ statistic for evaluation. Multi-CMDR is less sensitive to outliers and non-symmetric distributions.

While MDR is a nonparametric approach, all these methods use parametric test statistics as evaluation measures based on a multivariate normal distribution or exponential family distribution. Instead of using parametric approach, this study considers non-parametric evaluation measures for testing the equality of two multivariate populations. Various methods based on multivariate ranks or distances between the pairs of individual observations have been studied [25]. Note that signs and ranks in a univariate case are based on the ordering of the data. Unfortunately, however, there is no natural ordering of the data for a multivariate case. To extend the concept of rank to a multivariate case, several principles have been considered. First, the methods using interdirection were introduced [26, 27]. Interdirection is a measure based on the angular distance between two observation vectors relative to the rest of the data [28]. Second, the tests based on data depth were proposed [29–31]. A data depth measures how deep a multivariate sample lies in the data cloud [32]. Any function which provides a reasonable central-outward ordering of points in multidimensional space can be considered as a depth function [33]. Third, multivariate extensions using spatial signs and ranks were also studied [34, 35]. Affine invariant methods based on spatial sign and rank vectors for various multivariate problems were proposed [34]. More recently, for high-dimensional data, a nonparametric multivariate test using spatial signs [36] and a spatial ranks test for two samples were proposed [37]. Among various approaches and measures, we chose the spatial rank-sum statistic as the non-parametric evaluation measure in this study, because it is one of the most widely statistics and implemented with R program.

We propose a new non-parametric multivariate approach for identifying genetic interactions. We call the proposed method multivariate rank-based MDR (MR-MDR). During classification process, MR-MDR utilizes the fuzzy k-means clustering analysis with a noise cluster as in multi-CMDR. For the evaluation process, MR-MDR calculates the spatial rank-sum statistic as an evaluation measure and selects the best interaction model with the largest statistic. The tenfold CV method is adopted and the final best interaction model is determined by maximum CV consistency.

This manuscript is organized as follows. We first start with an introduction of the spatial rank statistic. The algorithm of the proposed MR-MDR method is then described in detail. We then present the results of intensive simulation studies to investigate the performance of the proposed method. Our method is compared with multi-QMDR and multi-CMDR. We applied the proposed MR-MDR method to data on four multivariate phenotypes related to kidney function obtained from the Korean Genome and Epidemiology Study (KoGES): blood urea nitrogen (BUN), serum creatinine, urinary albumin, and urinary red blood cell (RBC) levels. MR-MDR successfully identified genetic interactions associated with these four phenotypes.

## Methods

### Nonparametric multivariate rank test

We first introduce the multivariate non-parametric test used for evaluation. To detect a two-sample location shift in univariate analysis, the two-sample t-test is popularly used when the response variable is normally distributed. The Mann–Whitney test based on the rank sum is well known as a nonparametric counterpart of the two-sample univariate t-test. Various robust univariate non-parametric tests have been developed for the two-sample location problem [38].

For multivariate analysis, a classical approach such as Hotelling’s T^2^ is a popular parametric approach. T^2^ has optimal power under the assumption of a multivariate normal distribution. However, it performs poorly in the case of heavy-tailed distributions and is highly sensitive to outliers [39]. As an alternative, we consider a nonparametric approach based on spatial signs and ranks. We start with the definition of spatial sign and spatial rank.

Let ${\mathbf{Y}} = ({\mathbf{y}}_{{\mathbf{1}}} ,...,{\mathbf{y}}_{{\mathbf{n}}} )^{\prime }$ be an *n* × *p* data matrix with *n* observations and *p* variables. The spatial sign function and spatial rank function are defined as follows [28].

$$\begin{aligned} {\mathbf{S}}({\mathbf{y}}) & = \left\{ {\begin{array}{*{20}c} {||{\mathbf{y}}||^{ - 1} {\mathbf{y}},} & {{\mathbf{y}} \ne {\mathbf{0}}} \\ {{\mathbf{0}},} & {{\mathbf{y}} = {\mathbf{0}}} \\ \end{array} } \right., \\ {\mathbf{R}}({\mathbf{y}}) & = ave_{j} \{ {\mathbf{S}}{(}{\mathbf{y}} - {\mathbf{y}}_{{\text{j}}} {)}\} = \frac{1}{n}\sum\limits_{j = 1}^{n} {\{ {\mathbf{S}}{(}{\mathbf{y}} - {\mathbf{y}}_{{\text{j}}} } )\} \\ \end{aligned}$$

where *ave*_*j*_ means the average taken over all observations for j = 1,…n, ||**y**|| is the Euclidean distance of **y** from **0**, and **y** and **y**_*j*_ are p-variate vectors [28]. The observed spatial signs **s**_*i*_ and observed centered spatial ranks **r**_*i*_ are defined as

$${\mathbf{s}}_{i} = {\mathbf{S}}({\mathbf{y}}_{i} ),$$

and

$${\mathbf{r}}_{i} = ave_{j} \{ {\mathbf{s}}_{ij} \} = \frac{1}{n}\sum\limits_{j = 1}^{n} {\{ {\mathbf{S}}({\mathbf{y}}_{i} - {\mathbf{y}}_{j} )\} } ,$$

respectively for *i*, *j* = 1, …, *n*. Here, ${\mathbf{s}}_{ij} = {\mathbf{S}}({\mathbf{y}}_{i} - {\mathbf{y}}_{j} )$, *ave*{**r**_*i*_} = **0**.

To make an affine-invariant test statistic, we can apply the spatial sign function to the transformed data points. The test statistic $T({\mathbf{y}}_{1} ,...,{\mathbf{y}}_{n} )$ is said to be affine-invariant if $T({\mathbf{y}}_{1} ,...,{\mathbf{y}}_{n} ) = T(D{\mathbf{y}}_{1} ,...,D{\mathbf{y}}_{n} )$ for every *p* × *p* nonsingular matrix *D* and for every p-variate dataset ${\mathbf{y}}_{1} ,...,{\mathbf{y}}_{n}$ [34]. Affine-invariant spatial signs and ranks are obtained by transforming **y**_*i*_ to *A*_*y*_**y**_*i*_,

$$\begin{aligned} {\mathbf{s}}_{i}^{*} & = {\mathbf{S}}(A_{y} {\mathbf{y}}_{i} ), \\ {\mathbf{r}}_{i}^{*} & = ave_{j} \{ {\mathbf{s}}_{ij}^{*} \} = \frac{1}{n}\sum\limits_{j = 1}^{n} {\{ {\mathbf{S}}(A_{y} ({\mathbf{y}}_{i} - {\mathbf{y}}_{j} )\} } \\ \end{aligned}$$

where *A*_*y*_ is Tyler’s transformation, which makes the spatial sign covariance matrix proportional to the identity matrix, that is, $ave\{ {\mathbf{r}}_{i}^{{*{\prime }}} {\mathbf{r}}_{i}^{*} \} = [c_{y}^{2} /p]I_{p}$, where $c_{y}^{2} = ave\{ ||{\mathbf{r}}_{i}^{*} ||^{2} \}$. *A*_*y*_ can be obtained during the iterative process and chosen so that $trace(A_{y}^{\prime } A_{y} ) = p$ [34, 40, 41]. The ranks ${\mathbf{r}}_{i}^{*}$ lie in the unit p-sphere. The direction of ${\mathbf{r}}_{i}^{*}$ roughly points outward from the center of the data cloud and its length tells how far away this point is from the center of the data cloud [42].

Next, for the two-samples location problem, let ${\mathbf{Y}}_{1} = ({\mathbf{y}}_{1} ,...,{\mathbf{y}}_{{{\mathbf{n}}_{1} }} )^{\prime }$ and ${\mathbf{Y}}_{2} = ({\mathbf{y}}_{{{\mathbf{n}}_{1} + 1}} ,...,{\mathbf{y}}_{{{\mathbf{n}}_{1} + {\mathbf{n}}_{2} }} )^{\prime }$ be two independent samples with *p* variables that have the cumulative distribution functions $F({\mathbf{x}} - {{\varvec{\upmu}}})$ and $F({\mathbf{x}} - {{\varvec{\upmu}}} - {{\varvec{\Delta}}})$, respectively. To test the null hypothesis of no differences in location, *H*_0_:**Δ**:**0**, the multivariate version of Mann–Whitney test statistic *U*^2^ can be used. For the combined sample **Y** = [**Y**_1_:**Y**_2_], the affine-transformed spatial signs of the transformed differences ${\mathbf{s}}_{ij}^{*} = {\mathbf{S}}(A_{y} ({\mathbf{y}}_{i} - {\mathbf{y}}_{j} ))$ and spatial ranks ${\mathbf{r}}_{i}^{*} = ave_{j} \{ {\mathbf{S}}_{ij}^{*} \}$ are obtained. The multivariate Mann–Whitney test statistic *U*^2^ is given by

$$U^{2} = \frac{p}{{c_{y}^{2} }}\sum\limits_{i = 1}^{2} {n_{i} ||}^{{}} {\overline{\mathbf{r}}}_{i}^{*} ||^{2}$$

where ${\overline{\mathbf{r}}}_{i}^{*}$ is the sample-wise mean vector of the spatial ranks ${\mathbf{r}}_{i}^{*}$ and $c_{y}^{2} = ave\{ ||{\mathbf{r}}_{i}^{*} ||^{2} \}$ [34]. The p-value is obtained by $E_{\eta } (I(U_{\gamma }^{2} \ge U^{2} ))$, where $E_{\eta } ( \cdot )$ represents expectation value where $\eta = (\eta_{1} ,...,\eta_{N} )$ is uniformly distributed over *N*! permutations of $\left( {1, \ldots ,N} \right)$ and $U_{\gamma }^{2}$ is the value of the test statistic of a permuted sample. As the dimension *p* increases and the distribution becomes heavier-tailed, the performance of *U*^2^ improves relative to Hotelling’s *T*^2^ statistic [34].

**Fig. 1:**
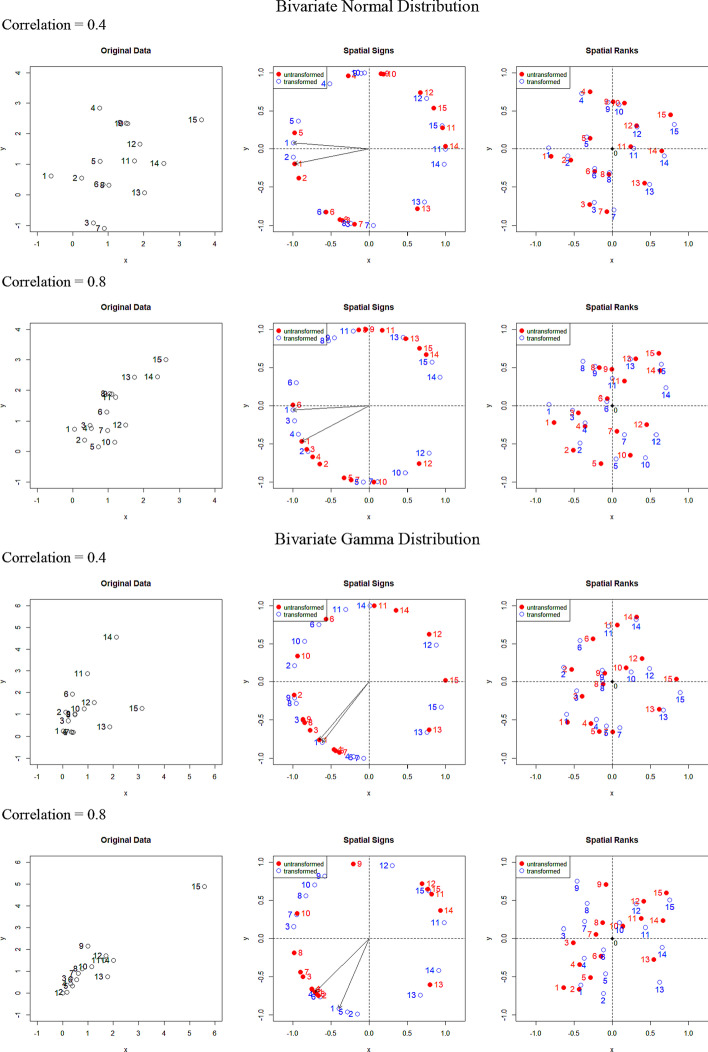
Examples of spatial signs and ranks for two bivariate distributions

We investigated the effect of Tyler’s transformation on **y**_*i*_ through an empirical study using a toy example. Two multivariate distributions of the response variables were considered: a bivariate normal distribution and a bivariate gamma distribution. The correlations between two response variables were set to 0.4 and 0.8. The original data were transformed to spatial signs, and then spatial centered ranks were obtained by averaging the spatial signs of differences. Figure 1 shows the spatial signs and ranks with and without Tyler’s transformation. Spatial signs are represented as directions from the origin with unit length, and thus all the spatial signs lie on a circle of radius 1. The spatial signs with Tyler's transformation tend to be more evenly arranged around the circumference than the spatial signs without Tyler's transformation. The spatial ranks tend to spread evenly, as if they are uniformly distributed within a circle, for the Tyler’s transformation case. Note that the spatial ranks before and after Tyler’s transformation appear different when the correlation is high (r = 0.8); however, the change due to the transformation is negligible if the correlation is moderate (r = 0.4).

### MR-MDR procedure

**Fig. 2:**
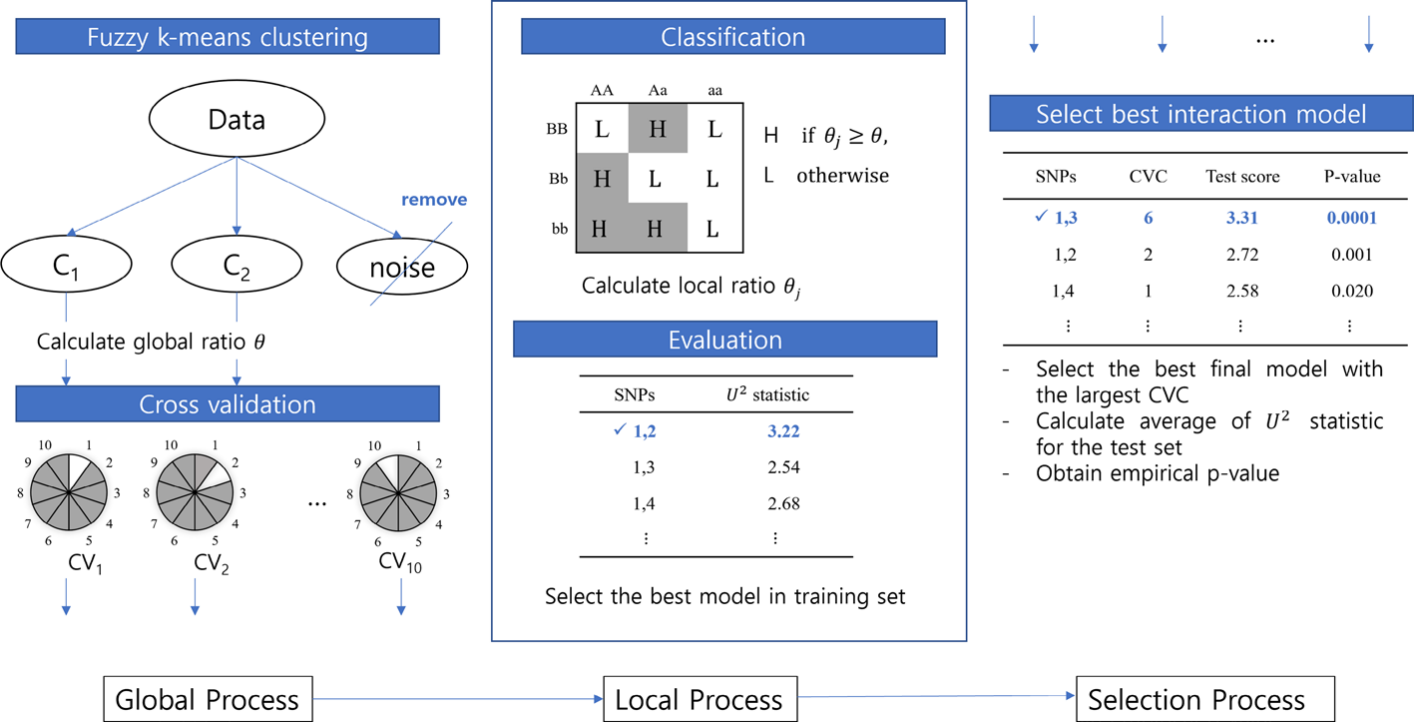
Overview of the MR-MDR algorithm for tenfold cross-validation and second-order interactions

There are many variations of MDR methods for finding GGIs. However, most MDR approaches have two core procedures: how to classify the cells into two groups and how to evaluate the interaction models. The proposed MR-MDR adopts fuzzy clustering technique in the classification process as in multi-CMDR [24]. For the evaluation process, MR-MDR uses a multivariate spatial rank test statistic. Figure 2 shows the flow of the MR-MDR procedure. The detailed algorithm of MR-MDR is as follows.

**Step 0. Data**

- Suppose there are n^*^ samples, with *p* SNP data-points and *q* continuous phenotypes.
- Standardize all the phenotypes to have a mean of zero and a unit variance. Let ${\mathbf{Y}}_{i} = (y_{i1} ,y_{i2} , \ldots ,y_{iq} )^{T}$ be the standardized phenotype vector and ${\mathbf{X}}_{i} = (x_{i1} ,x_{i2} , \ldots ,x_{ip} )^{T}$ be the genotype vector for the ith subject, respectively.

**Step 1. Global process: clustering**

- Perform fuzzy k-means clustering with k = 2 using phenotype information. We add a noise cluster such that noisy data points may be assigned to the noise class [43]. Remove all the samples in the noise cluster. The remaining samples have membership degrees for each of the two clusters. Denote these two clusters as *C*_1_ and *C*_2_.
- Define the global ratio *θ* as
  $$\theta = {{\sum\limits_{i = 1}^{n} {D_{i1} } } \mathord{\left/ {\vphantom {{\sum\limits_{i = 1}^{n} {D_{i1} } } {\sum\limits_{i = 1}^{n} {D_{i2} } }}} \right. \kern-\nulldelimiterspace} {\sum\limits_{i = 1}^{n} {D_{i2} } }},$$
  where *n* is the number of remaining samples after deleting noise samples and *D*_*ik*_ is the membership degree of the *i*th subject in cluster *C*_*k*_, (*k* = 1, 2) [24].
- For cross-validation (CV), split the samples randomly into 10 subgroups of equal size. Let nine sets of samples be the training dataset and the remaining dataset be the test dataset used for evaluating the model.

**Step 2. Local process: classification**

- To find *m*th-order GGIs, select a set of *m* SNPs from a pool of SNPs.
- Calculate the local ratio *θ*_*j*_ for the *j*th genotype combination in the training set,
  $$\theta_{j} = {{\sum\limits_{i = 1}^{{n_{j} }} {D_{ij1} } } \mathord{\left/ {\vphantom {{\sum\limits_{i = 1}^{{n_{j} }} {D_{ij1} } } {\sum\limits_{i = 1}^{{n_{j} }} {D_{ij2} } }}} \right. \kern-\nulldelimiterspace} {\sum\limits_{i = 1}^{{n_{j} }} {D_{ij2} } }},\quad \left( {j = 1, \ldots ,3^{m} } \right),$$
  where *D*_*ijk*_ is the membership degree of the *i*th subject with the *j*th genotype combination in cluster *C*_k_.
- Label each genotype combination either “H” if *θ*_*j*_ ≥ *θ*, or “L” if *θ*_*j*_ < *θ*.

**Step 3. Local process: evaluation**

- Obtain spatial ranks of *Y*_*i*_ for the combined samples from two clusters for *i* = 1, 2, …, *n*.
  $$\begin{aligned} r_{i}^{*} & = ave_{j} \{ {\mathbf{S}}(A_{y} ({\mathbf{y}}_{i} - {\mathbf{y}}_{j} )\} \\ & = \frac{1}{n}\sum\limits_{j = 1}^{n} {\frac{{A_{y} ({\mathbf{y}}_{i} - {\mathbf{y}}_{j} )}}{{\sqrt {(A_{y} ({\mathbf{y}}_{i} - {\mathbf{y}}_{j} ))^{2} } }}} \\ \end{aligned}$$
  where **S**(·)is a sign function and *A*_*y*_ is Tyler’s transformation.
- Calculate the multivariate Mann–Whitney test statistic:
  $$U^{2} = \frac{p}{{c_{y}^{2} }}\sum\limits_{k = 1}^{2} {n_{k} ||}^{{_{{}} }} {\overline{\mathbf{R}}}_{k} ||^{2}$$
  where *p* is the number of variables, *n*_*k*_ is the number of samples in cluster *C*_*k*_, ${\overline{\mathbf{R}}}_{k}$ is the mean vectors of the spatial ranks for cluster *C*_*k*_, and $c_{y}^{2} = ave\{ ||{\mathbf{R}}_{k} ||^{2} \}$.
- Obtain *U*^2^ for every *m* SNP combination. Choose the SNP combination with the largest *U*^2^ statistic as the best *m*th-order interaction model in the training data.

**Step 4. Selection process: best interaction model**

- Repeat step 2 and 3 for each fold and count the number of specific SNP combinations chosen for the best model. We call this cross-validation consistency (CVC).
- Select the model with the largest CVC as best final interaction model.
- Derive the final rank sum statistic for the best model by averaging all statistics for the test data.
- Calculate the empirical p-value by a permutation test.

## Results

### Simulation analysis

To compare the performance of the proposed MR-MDR with other existing methods, we conducted simulations for various situations. We considered 20 SNPs, including two-way disease-causal SNPs. For each of the combinations of seven different heritability values (0.01, 0.025, 0.05, 0.1, 0.2, 0.3, 0.4) and two minor allele frequency (MAF) values (0.2, 0.4), five different interaction models (M1-M5) were considered. Typically, penetrance rate is defined as the proportion of individuals with a given genotype who exhibit the phenotype associated with that genotype. However, it is not appropriate for continuous phenotypes and there is no clear definition of continuous phenotype. For QMDR, the penetrance for continuous phenotypes was defined as a function of mean [15]. Similarly, we set the penetrance rate as a function of mean of the response variable in each genotype. Thus, a total of 70 epistatic models with various penetrance functions were generated, as done by Velez et al. [7]. All the models had little marginal effect. For the phenotype distribution, we considered a bivariate normal distribution and a bivariate gamma distribution. The correlations of the bivariate phenotypes also varied (0, 0.25, 0.5). Sample sizes of 100, 200, and 400 were considered. Finally, a total of 1000 replicated data sets were generated for 1260 (= 70 × 2 × 3 × 3) combinations.

We used both a Tyler’s-transformed version (MR-MDR_transformed) and an untransformed version (MRMDR_ untransformed). For the purpose of comparison, multi-CMDR and multi-QMDR methods were also used. All three summary statistics of the multi-QMDR—the first principal component (FPC), weighted sum of principal components (WPC), and weighted squared sum of principal components (WSPC)—were included. To compare the data with the univariate approach, QMDR was also performed for each phenotype separately. Ten-fold CV was considered. A summary of the simulation scheme is shown in Table 1.

**Table 1:** Summary of the simulation scheme

	Factor	Level	Value	Ref
Genotype	Number of SNPs	20	SNP_1_, …, SNP_20_	
	Number of causal SNPs	2	SNP_1_, SNP_2_	
	Minor allele frequency	2	0.2, 0.4	
	Heritability	7	0.01, 0.025, 0.05, 0.1, 0.2, 0.3, 0.4	
	Interaction model	5	M1, M2, M3, M4, M5	[7]
Phenotype	Number of phenotypes	2	Y_1_, Y_2_	
	Distributions	2	bivariate normal, bivariate gamma	[24, 44]
	Correlation	3	0, 0.25, 0.5	
Sample	Sample size	3	100, 200, 400	
Analysis	Methods		MR-MDR (transformed, untransformed)	[24]
		8	multi-CMDR	[23, 24]
			multi-QMDR (FPC, WPC, WSPC)	[15]
			QMDR (Y_1_, Y_2_)	

*FPC* first principal component, *WPC* use weighted Summation of principal component, *WSPC* use weighted Squared Summation of principal component

Since the epistatic models given by Velez et al. [7] were devised only for the discrete phenotype, we modified them to handle continuous phenotypes. Let ${\mathrm{SNP}}_{1}$ and ${\mathrm{SNP}}_{2}$ be the two true causal SNPs, ${\mathbf{Y}} = (Y_{1} ,Y_{2} )^{T}$ the continuous bivariate phenotypes, and *f*_*ij*_ the penetrance function for the ith genotype of ${\mathrm{SNP}}_{1}$ and jth genotype of ${\mathrm{SNP}}_{2}$ in [7]. For the bivariate normal distribution, ${\mathbf{Y}} = (Y_{1} ,Y_{2} )^{T}$ was generated by

$${\mathbf{Y}}|({\text{SNP}}_{{1}} = i,{\text{ SNP}}_{2} = j)\sim MN({{\varvec{\upmu}}}_{ij} ,{{\varvec{\Sigma}}}),$$

where $${{\varvec{\upmu}}}_{ij} = \left( {\begin{array}{*{20}c} {f_{ij} } \\ {f_{ij} } \\ \end{array} } \right)$$ and $${{\varvec{\Sigma}}} = \left( {\begin{array}{*{20}c} 1 & \rho \\ \rho & 1 \\ \end{array} } \right)$$. We used the mvrnorm() function of the MASS package in R. Three different values of *ρ* (0, 0.025, and 0.5) were considered, as mentioned above.

For the skewed asymmetrically distributed phenotypes, we used the copula-based multivariate gamma model [44]. A copula-based bivariate gamma distribution is given by

$$f(y_{1} ,y_{2} ) = c(u,{{\varvec{\Sigma}}})\prod\limits_{k = 1}^{2} {f_{k} (y_{k} )} ,$$

where $c(u,{{\varvec{\Sigma}}}) = |{{\varvec{\Sigma}}}|^{\frac{1}{2}} \exp \left[ { - \frac{{{\tilde{\mathbf{u}}}^{{\prime }} ({{\varvec{\Sigma}}}^{ - 1} - {\rm I}){\tilde{\mathbf{u}}}}}{2}} \right],\tilde{u} = (\Phi^{ - 1} (u_{1} ), \, \Phi^{ - 1} (u_{2} ))^{{\prime }}$, and $u_{k} = {\mathbf{F}}_{k} (y_{k} )$ for k = 1, 2. Here *f*_*k*_ and **F**_*k*_ are the marginal probability density function and cumulative distribution function of the kth phenotype, respectively, and Φ^−1^ is the inverse of the cumulative distribution of the standard normal distribution function. In this simulation, we set

$$y_{k} |({\text{SNP}}_{1} = i,{\text{ SNP}}_{2} = j)\sim Gamma(2f_{ij} ,0.5)$$

for k = 1, 2. We used the mvdc(), rMvdc(), normalCopula() functions in the copula package in R.

**Fig. 3:**
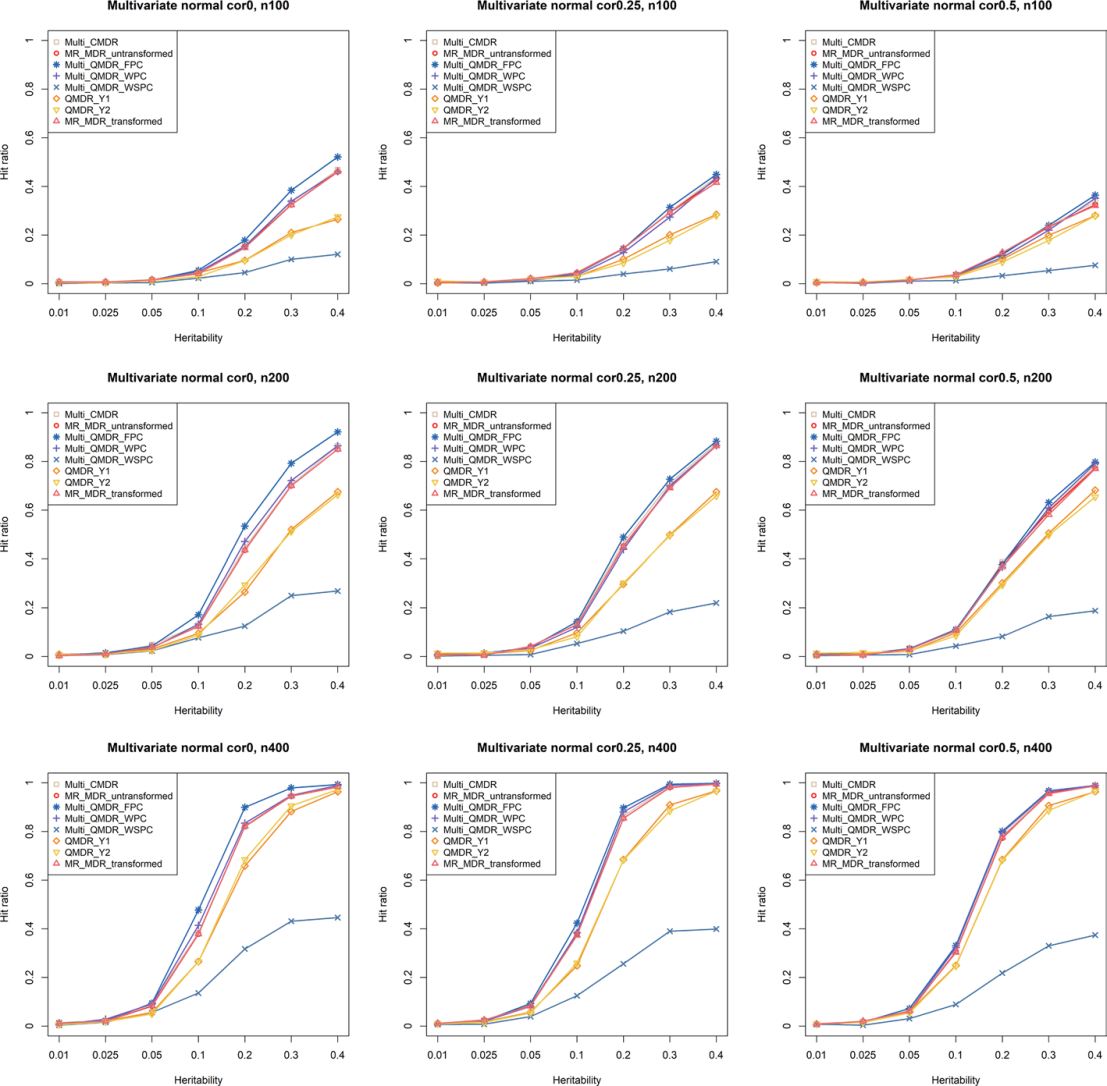
Hit ratios for a bivariate normal distribution

The power was estimated by the hit ratio, which is the proportion of times that each method correctly chose the causal SNP pairs (${\mathrm{SNP}}_{1}$ and ${\mathrm{SNP}}_{2}$) as the best model among all possible two-way interaction models out of each set of 1000 datasets. Figure 3 shows the hit ratios of the eight different methods for the bivariate normal distribution. The power of the multi-QMDR using FPC (multi-QMDR_FPC) was slightly higher than that of the proposed MR-MDR when there was no correlation between phenotypes. However, the difference between multi-QMDR_FPC and MR-MDR decreased as the correlation became stronger. The performances of the transformed one (MR-MDR_transformed) and untransformed (MR-MDR_untransformed) one were almost the same. Multi-QMDR with WSPC (multi-QMDR_WSPC) showed lower power even than the QMDR method.

**Fig. 4:**
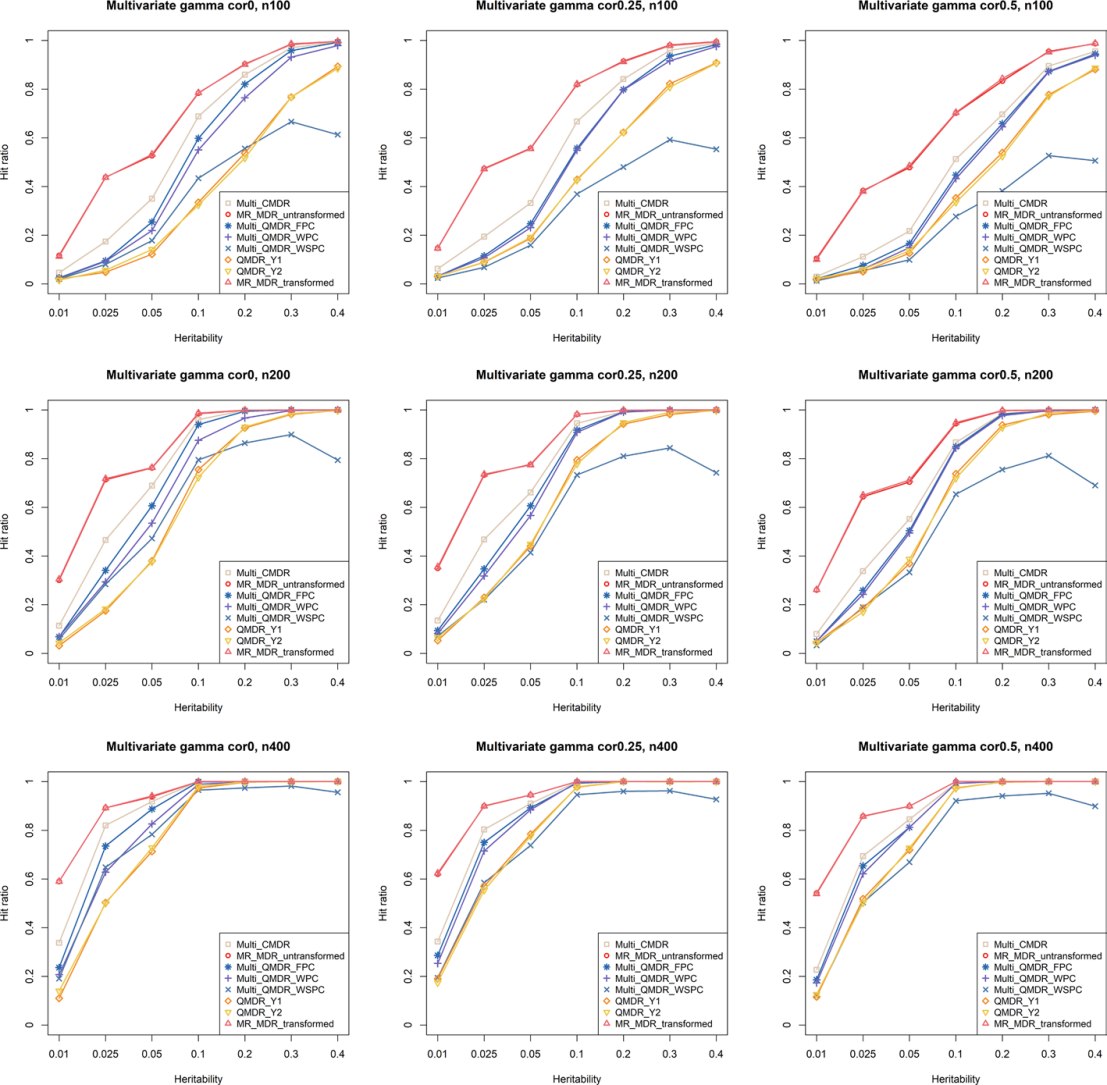
Hit ratios for a bivariate gamma distribution

Figure 4 shows the hit ratios for a bivariate gamma distribution. The proposed MR-MDR outperformed the other methods for all ranges of heritability. There was little difference between the performance of the two versions of MR-MDR, and the differences between them were less than 0.01 in all situations. The power of multi-CMDR was the next highest. It should be noted that multi-CMDR uses the fuzzy clustering approach to split data as in MR-MDR. The gap between MR-MDR and other methods became larger as the sample size decreased or the correlation became stronger. Multi-QMDR-FPC and multi-QMDR using the WPC (multi-QMDR-WPC) showed lower power than MR-MDR and multi-CMDR, but higher power than QMDR. The performance of multi-QMDR-WSPC was still poor, although the difference was less than in bivariate normal distribution.

Through these simulation studies, we demonstrated that the proposed MR-MDR outperformed the other existing methods for all ranges of heritability when the phenotypes were asymmetrically distributed. However, when the phenotypes are symmetrically distributed, as in a normal distribution, all methods yielded similar performance.

Three additional simulations were conducted to find out the further properties of the proposed method. The robustness of fuzzy k-means clustering, the effect of noise cluster size, and the effect of outliers were investigated. First, to explore the robustness of the fuzzy k-means clustering in MR-MDR algorithm, we performed a comparison study to investigate the effect of log-transformation on phenotypes which were generated from the bivariate gamma distribution. The power of MR-MDR after log transformation was obtained for each seven heritabilities. We set the correlation coefficient between two phenotypes 0.25 and the sample size 200. The average power of MR-MDR for five interaction model (M1-M5) for 1000 random samples are given in Table 2. The power slightly reduced after log-transformation. Therefore, we can conclude that the fuzzy k-means clustering is robust to the skewed distribution and does not require any further transformation of original data.

**Table 2:** Hit ratios of MR-MDR according to log transformation for a bivariate gamma distribution (r = 0.25, n = 200)

Heritability	Hit ratio	
	Without log transformation	With log transformation
0.01	0.350	0.325
0.025	0.733	0.685
0.05	0.776	0.693
0.1	0.982	0.942
0.2	0.999	0.989
0.3	1	0.999
0.4	1	1

Secondly, we investigate the efficiency according to the size of noise cluster since the large size of the noise cluster can be a source of loss of efficiency. The size of noise cluster depends on the noise distance *δ*, which needs to be chosen in advance. If *δ* is too large, outliers are not removed and are classified as a real cluster. On the other hand, if *δ* is too small, many observations can be misplaced into the noise cluster. Still, the estimation of the optimal value of *δ* is an open-problem [45]. In our approach, we used an iterative procedure to determine the value of *δ* optimally, which is the default of FKM.noise procedure in R.

To check the efficiency, we compared the performance with various values of *δ*. The results are shown in Table 3. For both bivariate normal and bivariate gamma distribution, the average power of MR-MDR for five interaction models (M1-M5) are given in Table 3. This new simulation result shows that the power using the iterative *δ* yielded the highest hit ratios for all heritabilities. Note that in this setting outliers were not generated. When there were outliers, a smaller noise cluster would have been created.

**Table 3:** Hit ratios of MR-MDR according to the noise distance *δ* (r = 0.25, n = 200)

Distribution	Heritability	$\delta$= 1.5		$\delta$= 2		$\delta$= 2.5		Iterative $\delta$ (default)	
		Hit ratio	Noise (%)	Hit ratio	Noise (%)	Hit ratio	Noise (%)	Hit ratio	Noise (%)
Bivariate	0.01	0.004	14.78	0.007	5.10	0.005	1.27	0.009	12.02
Normal	0.025	0.003	14.78	0.008	4.96	0.007	1.16	0.006	11.90
	0.05	0.024	14.79	0.026	5.04	0.038	1.19	0.041	11.92
	0.1	0.073	14.54	0.1	4.86	0.131	1.12	0.131	11.59
	0.2	0.255	14.27	0.372	4.69	0.423	1.10	0.452	11.21
	0.3	0.430	14.28	0.609	4.70	0.669	1.08	0.693	11.14
	0.4	0.593	13.944	0.793	4.54	0.846	1.03	0.865	10.736
Bivariate	0.01	0.302	11.89	0.335	7.47	0.352	4.88	0.35	8.25
Gamma	0.025	0.666	11.734	0.709	7.42	0.718	4.84	0.733	7.98
	0.05	0.714	12.67	0.738	7.50	0.758	4.62	0.776	8.93
	0.1	0.955	13.09	0.972	7.36	0.978	4.25	0.982	9.25
	0.2	0.995	13.00	0.996	6.86	0.998	3.68	0.999	9.17
	0.3	0.998	12.47	1	6.67	1	3.59	1	8.65
	0.4	1	12.18	1	6.30	1	3.37	1	8.27

Thirdly, we have conducted an additional simulation to investigate the effects of outliers. The power of MR-MDR for the datasets with or without outliers was obtained for each seven heritabilities. We set the correlation coefficient between two phenotypes 0.25 and the sample size 200 as in Table 3. The power for five interaction models (M1-M5) for 1000 random samples were obtained. For the datasets with outliers, about 5% of the data were replaced by outliers. For both phenotypes, outliers were generated by adding three times of IQR for the randomly chosen 5% samples. The results are summarized in Table 4. In the presence of outliers, the power tends to decrease for all methods. The differences were the smallest in MR-MDR, indicating the minimum power loss of MR-MDR. As a result, MR-MDR showed much higher power than other methods in the presence of outlying observations.

**Table 4:** Hit ratios of MR-MDR according to the presence or absence of outliers (r = 0.25, n = 200)

	Heritability	MR-MDR			Multi-CMDR			Multi-QMDR_FPC		
		WO	W	Diff	WO	W	Diff	WO	W	Diff
Bivariate	0.01	0.009	0.006	0.003	0.006	0.006	0	0.009	0.007	0.002
Normal	0.025	0.006	0.006	0	0.007	0.006	0.001	0.014	0.013	0.001
	0.05	0.041	0.033	0.008	0.039	0.034	0.005	0.036	0.018	0.018
	0.1	0.131	0.09	0.041	0.142	0.092	0.05	0.143	0.038	0.105
	0.2	0.452	0.364	0.088	0.465	0.375	0.09	0.489	0.121	0.368
	0.3	0.693	0.604	0.089	0.711	0.593	0.118	0.727	0.251	0.476
	0.4	0.865	0.781	0.084	0.873	0.79	0.083	0.883	0.353	0.53
Bivariate	0.01	0.35	0.26	0.09	0.135	0.096	0.039	0.094	0.072	0.022
Gamma	0.025	0.733	0.609	0.124	0.468	0.355	0.113	0.347	0.276	0.071
	0.05	0.776	0.707	0.069	0.661	0.494	0.167	0.606	0.446	0.16
	0.1	0.982	0.947	0.035	0.945	0.748	0.197	0.917	0.692	0.225
	0.2	0.999	0.992	0.007	0.994	0.934	0.06	0.993	0.82	0.173
	0.3	1	1	0	1	0.989	0.011	0.999	0.929	0.07
	0.4	1	1	0	1	0.999	0.001	0.999	0.974	0.025

*W/O* hit ratio for the data without outlier, *W/* hit ratio for the data with outlier, *Diff* W/O – W, *Multi-QMDR_FPC* multi-QMDR using first principal component

### Real data analysis

We illustrate the proposed MR-MDR method by analyzing data from the KoGES [46]. The data were collected from two recruitment areas. Each region is a cohort representing city (Ansan) and rural areas (Anseong). After standard quality control procedures for the subjects and SNPs, a total of 8842 participants and 327,872 SNPs were used for this analysis.

We considered four phenotypes related to kidney function: BUN, serum creatinine, urinary albumin levels, and urinary RBC levels. Traditionally, BUN and serum creatinine levels have been used as surrogate markers of kidney function deterioration [47]. The amounts of albumin and RBC in urine also could be good indicators of kidney disease. Although there have been some studies on associations between genes and kidney-related phenotypes, few studies have performed GGI analyses for these phenotypes [48, 49]. In the case of albumin, the urine test is known to be more accurate than in the case of blood test, so the urine test result is used here. However, urine tests are conducted only on a relatively small number of subjects, which resulted in missing observations in phenotypes. For this high rate of missing data, imputation for phenotypes is not appropriate. We removed observations with at least one missing phenotype value, and 3267 samples remained.

**Fig. 5:**
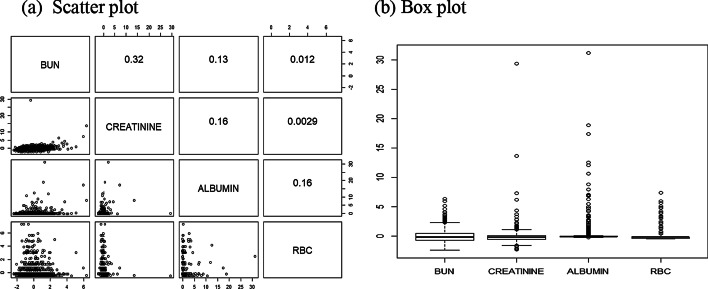
Scatterplot and boxplot of four phenotypes after adjustment by sex, age and recruitment area. The numbers in the scatter plot are correlation coefficients

A linear model was separately fit to each phenotype with covariate adjustments for sex, age and recruitment area. Finally, 205 SNPs were selected that had significant marginal effects (*p* < 1 × 10^−7^). Residuals were used for the analysis to control for covariate effects. The largest correlation coefficient was 0.32, which was the correlation between BUN and creatinine. The distributions of the phenotypes were heavily skewed. Figure 5 shows scatter plots and box plots of the phenotypes.

We applied the proposed MR-MDR method to identify the best first- and second-order interaction models. Table 5 shows the center of the global clusters from fuzzy k-means clustering during step 1. Cluster centers were determined by the means of each phenotype across samples belonging to the cluster. The clusters differed greatly for BUN and serum creatinine. There seemed to be no difference in RBC levels between the two clusters. Since the higher values of BUN and creatinine indicate abnormal kidney function, we can interpret the first cluster as a low-risk group and the second cluster as a high-risk group.

**Table 5:** Average of phenotypes for global clusters

	Cluster 1 (L)	Cluster 2 (H)	All
BUN	13.55	16.27	11.06
Serum creatinine	0.78	0.84	0.72
Albumin	2.66	3.39	1.99
RBC	0.20	0.21	0.20
Number of cases	1410	1225	2635

We selected the SNP pair with the largest CVC as the best interaction model for each order. The test score was the average of spatial rank sum statistics calculated from the test data set. Empirical p-values were obtained by comparing the test score with the statistic from the permuted dataset generated by shuffle phenotype vectors. If the score calculated with the permuted data exceeded our score, that case was counted. Then p-value was calculated as *a*/*b*, where *a* is the number of cases with a permuted score higher than the original score and *b* is the total number of trials.

List of the interaction models that had the highest training score at least once during the tenfold CV process by MR-MDR is shown in Table 6. For the first-order interaction, rs41476549 had the highest CVC and was selected as the most relevant to the four phenotypes. rs790410 was selected as the best once during 10 CV processes but showed the highest score. All SNPs except rs17168600 had a p-value lower than 0.05. For the second-order interaction model, the pair of rs41476549 and rs1117105 showed the highest CVC, while the pair of rs790410 and rs961413 yielded the highest test score. However, the CVC value was low in most cases. Among the selected SNPs, rs16862782 on chromosome 3 has been reported to be related to BUN in Korean and to serum urea measurement in European [47, 50]. rs4686914 on chromosome 3 is also known to be related to the kidney function in European and East Asian [47, 51]. Both SNPs are mapped to LINC01991 gene. rs17586294 maps to TUBBP11 gene. To the best of our knowledge, there are no studies that have analyzed kidney-related GGI in a multivariate manner. Therefore, none of the interactions of the selected pair of SNPs have ever been reported.

**Table 6:** Best interaction models identified by MR-MDR, multi-CMDR and multi-QMDR

Method	First order				Second order			
	rs ID	CVC	Test Score	p-value	rs ID	CVC	Test Score	p-value
MR-MDR	rs41476549	5	5.05	0.022	rs41476549, rs1117105	2	6.23	0.015
	rs790410	1	6.20	0.003	rs790410, rs961413	1	7.31	0.002
	rs16862782	1	5.17	0.015	rs11250624, rs17168600	1	7.21	0.003
	rs1348637	1	4.99	0.027	rs41476549, rs790410	1	7.01	0.004
	rs291877	1	4.94	0.029	rs17168600, rs41476549	1	6.94	0.004
	rs17168600	1	4.39	0.064	rs291877, rs790410	1	6.48	0.013
					rs7706475, rs41476549	1	6.20	0.016
					rs4686914, rs16992376	1	3.38	0.583
					rs17586294, rs7016675	1	3.33	0.601
Multi-CMDR	rs1348637	3	1.32	0.062	rs790410, rs961413	2	1.87	0.003
	rs16862821	2	1.23	0.110	rs291877, rs7612956	1	1.93	0.002
	rs790410	1	1.57	0.012	rs291877, rs17168600	1	1.73	0.007
	rs291877	1	1.54	0.015	rs291877, rs961413	1	1.65	0.012
	rs4686914	1	1.45	0.027	rs291877, rs17643945	1	1.45	0.038
	rs41476549	1	1.17	0.162	rs17142042, rs1160759	1	1.19	0.002
	rs17142042	1	0.89	0.538	rs17643945, rs291877	1	1.45	0.038
					rs12337165, rs9958995	1	1.01	0.364
					rs9812308, rs12860002	1	0.78	0.733
Multi-QMDR_FPC	rs10517358	6	27.05	< 0.001	rs17014894, rs10517358	6	51.05	< 0.001
	rs7265852	3	2.86	0.002	rs41457747, rs10517358	2	27.58	< 0.001
	rs17014894	1	24.17	< 0.001	rs41457747, rs17014894	1	24.35	< 0.001
					rs1631834, rs10517358	1	2.97	< 0.014

We also applied multi-CMDR and multi-QMDR methods for comparison. Only multi-QMDR using FPC was considered for comparison, because it showed the highest power among three types of multi-QMDRs. There are some similarities between the results of MR-MDR and multi-CMDR. However, the results are totally different for multi-QMDR, which are expected to be caused by some outlying observations. For example, the pair of rs17014894 and rs10517358 was chosen as the best interaction model. However, this pair suffers from sparsity and outliers. In particular, there are four cells with zero counts and one cell with one count having an outlying observation.

**Fig. 6:**
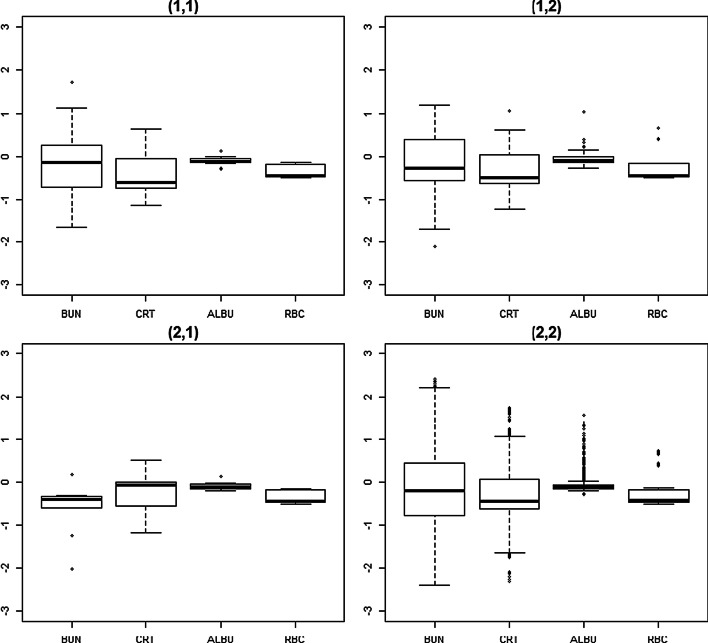
Box plots of four phenotypes after removing the noise cluster for the best SNP combination identified by MR-MDR ((i, j): ith genotype for rs1117105 and jth genotype for rs41476549, *s* creatinine, *ALBU* albmin)

Figure 6 shows the box plots of the phenotypes after removing the noise cluster for the genotype combinations of the best model selected by MR-MDR. The distribution of each phenotype was different depending on the genotype combination, suggesting that there was an interaction effect.

To evaluate pure interaction effects for continuous phenotypes, we adopted the classical linear model. For the selected SNP combinations, we fit multivariate analysis of variance (MANOVA) model including two main effects and the interaction effect. The SNP effects were tested for the interaction effect only (*H*_01_:*β*_12_ = 0) and for the total effects including both main and interaction effects (*H*_02_:*β*_1_ = *β*_12=_0 and *H*_03_:*β*_2_ = *β*_12=_0). The results nine SNP pairs are summarized in Table 7. None of the selected SNP pairs showed significant interaction effects (p > 0.05), while some pairs showed significant total effects. This is because most MDR methods tend to choose a model with a large total effect ignoring pure interaction effects. However, our further empirical study showed that the introduction of noise cluster by fuzzy k-means increased the power of detecting interaction effects. The same MANOVA model were fit to the trimmed data after removing the noise cluster by MR-MDR. As expected, several significant interaction effects were successfully identified after trimming. Although MANOVA requires a Gaussian assumption, the process removing the noise cluster in the proposed method had the effect of yielding more robust and reliable MANOVA results. Among the nine SNP pairs with non-significant interactions, four pairs showed significant interaction effects. The results are summarized in the last three columns of Table 7.

**Table 7:** p-values from MANOVA test for the SNP combination selected by MR-MDR

rs ID	Original data (including noise)			Trimmed data by MR-MDR (excluding noise)		
	${H}_{01}$^*^	${H}_{02}$^**^	${H}_{03}$^***^	${H}_{01}$^*^	${H}_{02}$^**^	${H}_{03}$^***^
rs41476549, rs1117105	0.6971	0.5841	0.2237	0.9209	0.2201	0.1516
rs790410, rs961413	0.6887	0.00001	0.4374	0.4077	0.0546	0.0140
rs11250624, rs17168600	0.4574	0.0000002	0.5569	0.0388	0.0258	0.0027
rs41476549, rs790410	0.7868	0.2603	0.6852	0.7505	0.1327	0.0970
rs17168600, rs41476549	0.7221	0.7838	0.1680	0.4196	0.0704	0.0477
rs291877, rs790410	0.2365	0.0065	0.0543	0.0107	0.0009	0.0009
rs7706475, rs41476549	0.2289	0.5622	0.0586	0.0211	0.0278	0.0024
rs4686914, rs16992376	0.6290	0.0008	0.8686	0.8155	0.0004	0.7291
rs17586294, rs7016675	0.8552	0.6386	0.8003	0.0003	0.0007	0.0011

^*^H_01_:*β*_12=_0

^**^H_02_:*β*_1_ = *β*_12=_0

^***^H_03_:*β*_2_ = *β*_12=_0

## Discussion

The proposed MR-MDR method is a non-parametric approach assuming no particular genetic model. To assign samples to two risk groups, MR-MDR uses the fuzzy clustering technique, as in the multi-CMDR method. MR-MDR uses the spatial rank sum statistic as evaluation measure for comparing two groups whereas Hotelling’s T^2^ statistic is used in multi-CMDR and multi-QMDR. Therefore, robust results can be expected in MR-MDR for skewed distributions or those with outliers.

When calculating the spatial rank statistic, a data-driven transformation matrix was needed to make the statistic invariant. It is known that an affine-invariant test performs better than noninvariant angle coordinate-wise sign tests when there is significant deviation from spherical symmetry caused by the presence of correlations among observed variables. Moreover, the affine-invariant test outperforms Hotelling’s T^2^-test for heavy-tailed non-normal multivariate distributions [52]. As can be seen in our toy example, the invariant statistic differed from the untransformed statistic, especially in the presence of a high correlation between phenotypes.

The problem with using Tyler's transformation statistic is that it takes much longer to calculate than the untransformed statistic. However, the change of the spatial ranks due to the Tyler’s transformation is trivial even when the correlation is moderate (r = 0.4), as seen in the toy example. Moreover, the simulation results showed that there was little improvement in performance compared to untransformed versions of MR-MDR. This phenomenon was also observed in the case of negative correlation. Therefore, we recommend using the untransformed MR-MDR version if the absolute value of correlation between phenotypes is not too high (e.g., |r|< 0.5).

To apply the proposed method for GWAS data, we considered a filtering procedure to reduce the number of SNPs to be investigated. We selected SNPs with significant marginal effects and investigated the interactions. Since MDR is an exhaustive method, this kind of filtering is needed. However, this filtering process can lead to ignoring gene–gene interactions for the SNPs with weak marginal effects.

## Conclusions

In this paper, we proposed the MR-MDR method to detect the best interaction model for multivariate quantitative traits. MR-MDR is based on the fuzzy clustering technique and spatial rank-sum statistic. Intensive simulation studies comparing MR-MDR with several current methods showed that the performance of MR-MDR was outstanding for skewed distributions. The difference in power between the MR-MDR and other methods increased as the sample size became smaller and the correlation became stronger. Additionally, for symmetric distributions, MR-MDR showed comparable power. Therefore, we conclude that MR-MDR is a useful multivariate non-parametric approach that can be used regardless of the phenotype distribution, the correlations between phenotypes, and sample size.

## Data Availability

The Korea Association Resource (KARE) project data will be publicly distributed by the Distribution Desk of Korea Biobank Network (https://koreabiobank.re.kr/). The data request should be made directly to Distribution Desk of Korea Biobank Network. Any inquiries should be sent to admin@koreabiobank.re.kr.

## Abbreviations

GGI: Gene–gene interaction
MDR: Multifactor dimensionality reduction
CV: Cross validation
CVC: Cross validation consistency
SNP: Single nucleotide polymorphism
BMI: Body mass index
BUN: Blood urea nitrogen
RBC: Red blood cell
MAF: Minor allele frequency
FPC: First principal component
WPC: Weight sum of principal components
WSPC: Weighted squared sum of principal components
MANOVA: Multivariate analysis of variance
